# A Qualitative Exploration of Chinese Self-Love

**DOI:** 10.3389/fpsyg.2021.585719

**Published:** 2021-03-29

**Authors:** Li Ming Xue, Xi Ting Huang, Na Wu, Tong Yue

**Affiliations:** Faculty of Psychology, Southwest University, Chongqing, China

**Keywords:** self-love, qualitative study, Chinese, public view, connotation, structure

## Abstract

Although self-love is an important topic, it has not been viewed as appropriate for psychological research, especially in China. We conducted two studies to understand how Chinese people view self-love. In the first study, we surveyed 109 Chinese people about the dimensions of self-love using an open-ended questionnaire. In the second study, 18 participants were selected by means of intensity sampling and interviewed about the connotations and structure of Chinese self-love. The two studies revealed three important aspects of the Chinese understanding of self-love: (1) self-love has four dimensions: self, family, others, and society; (2) it comprises five components: self-cherishing, self-acceptance, self-restraint, self-responsibility, and self-persistence; and (3) the five components of self-love are linked together to form a stable personality structure. The reliability and validity of the two studies were strong. Finally, the results showed that Chinese self-love is dominated by Confucian culture, which provides guiding principles for how to be human. At the same time, it shows that there are differences in the understanding of self-love between Chinese and Western cultures, which provides an empirical basis for further research based on cross-cultural psychology and self-love psychology.

## Introduction

*In our search for self-love, we have only reached the discovery that self-love remains an unknown world to us*.

—François de La Rochefoucauld

Self-love is an important aspect of human life with personal and moral significance. It enhances health and longevity, prompting individuals to strive for their own good and perfection (Rocha and Ghoshal, 2006; Gebauer et al., 2012); moreover, it enables individuals to conform to social norms and is even regarded as an important force in the fight against terrorism (Kruglanski et al., 2013). In psychology, researchers have viewed this important topic in different ways. Some researchers saw self-love as self-feelings that comprise four self-relevant emotions (ashamed, humiliated, proud, and pleased), also known as affective self-regard/self-love (Cai et al., 2007; Brown, 2010). From this perspective, studies have found that, when using implicit measures, cross-cultural differences in self-esteem and self-enhancement tend to disappear (Kitayama and Uchida, 2003; Kobayashi and Greenwald, 2003; Cai, 2006; Yamaguchi et al., 2007). Based on this, it has been suggested that self-love may be a fundamental, implicit human motive to fit into a specific cultural role (Leary and Tangney, 2012). However, some researchers have regarded self-love as a positive way of treating oneself that remains stable over time and across situations. For example, Fromm (1947) put forward that self-love is to love oneself; to love oneself is to care for oneself, be responsible for oneself, respect oneself, and understand oneself. Freud (1957, p. 67) believed that self-love was a form of narcissism, where the libido was directed toward oneself and inversely proportional to the love felt for others. Benjamin et al. (2006) considered self-love to be a gentle love for oneself that focused on the cultivation, care, and development of oneself. However, Chinese scholars believed that self-love included not only taking good care of one's body but also cherishing one's reputation and paying attention to one's words and deeds—in other words, to please, cherish, and respect one's own body, character, ability, reputation, position, and future (Lin et al., 2003; Yang, 2009, p. 66; Huang, 2017, p. 56). It is apparent that Western and Chinese scholars have different views regarding self-love. Western scholars' description of self-love is relatively broad, while Chinese scholars' description of self-love is more specific, including body, ability, reputation, etc., and has the meaning of restraint. According to this notion, just as people who love themselves will not do anything harmful to their body, so will they not harm their reputations.

The difference between the manner in which Chinese and Western psychologists understand self-love may be due to their respective cultural backgrounds. In ancient Greek philosophy, *oikeiosis* (self-love) was considered the source of all good and radiated a circle of love, first for oneself, then for one's children, then for one's family, and even for all humanity. According to Aristotle, self-love was the core of friendship; a person should be their own best friend first, then extend love to others. The ancient Greek philosophers regarded self-love as one's relationship with oneself and as a person's inner sense of value. Therefore, it was considered to be a basic moral relationship (Chazan, 1998). During the Spring and Autumn period of Chinese history, scholars such as Confucius and Mencius regarded self-love as both the starting point and the acme of *Ren* (one's natural virtues). They stated: “The man of virtue, while establishing himself and pursuing success, also works to establish others and enable them to succeed as well” (Chen and Xu, 2015); “Love, from self-love; and *Ren*, from *Ren*. To be kind to people, and then to love living things. It can be said to be *Ren* if the three are consistent” (Mencius one); “You can be called a wise *Junzi* (a man of noble character), if you can respect and love yourself” (Fang and Li, 2015). Therefore, self-love is “everyone has the virtue of a *Junzi*” (Fang and Li, 2015; Xue et al., 2020) as a foothold and “considers others in one's own place.”

Although Chinese and Western philosophers believe that self-love is a virtue, their function orientation is different. In the Western philosophical context, self-love is concerned with the individual's rights, dignity, and intrinsic sense of value, as well as one's relationship with oneself, and society as a whole only serves to promote an individual's happiness. In the context of Chinese philosophy, self-love focuses more on the individual's conduct and ethics, and the peace, harmony, and order of the whole society depend on the individual's self-cultivation (Thompson and Tu, 1987). This difference in the philosophical background underlies the cultural differences in the understanding of self-love between Chinese and Western cultures. Some studies by Western scholars of specific groups (e.g., African-American girls, female college students who have experienced sexual violence, or people living with AIDS) have found a variety of manifestations of self-love, such as self-confidence, self-acceptance, participation in self-care activities, physical recovery, challenging negative self-concepts (Phelps-Ward and Laura, 2016; Lahad and Kravel-Tovi, 2019; Sinko et al., 2019; Tokwe and Naidoo, 2020). In the context of Chinese culture, Xue et al. (2020) conducted a psychological analysis of self-love in the Siku Quanshu (the most systematic and comprehensive summary of the official books of classical Chinese culture). The results showed that self-love, as understood by the ancient Chinese, included three levels: personal self-love (self boundary), individual self-love (family boundary), and social self-love (country and world boundary). It also included three components: self-cherishing (cherish and care for one's life, body, reputation, property, monarchy, country, people, and so on), self-acceptance (be able to accept oneself under any realistic conditions), and self-restraint (restrain oneself with law and morality and be careful in words and deeds). To some extent, the above results reflect the differences in people's understanding of self-love in different cultural backgrounds.

Unfortunately, self-love has not been regarded as an appropriate research topic. Especially in the context of Chinese culture, self-love research is rare. Considering this, how do modern Chinese adults understand self-love? What does self-love involve? What are the components of its personality structure? These are the questions we sought to answer in this study. According to Chen (2000, p. 12), qualitative research is

*a kind of activity that takes the researcher as the research tool, uses various kinds of data collection methods in the natural situation to carry on the overall research to the social phenomenon, uses the induction method to analyze the data and the formation theory, obtains the explanatory understanding through the interaction with the research object to its behavior and the meaning construction*.

Qualitative research is a tool used to describe and understand the world of human experiences. To comprehend Chinese people's understanding of self-love in both breadth and depth, to provide a reference point for psychological research into self-love, and to provide evidence for the differences in the understanding of self-love in cross-cultural contexts, we conducted two qualitative studies in the context of Chinese culture. First, we conducted a survey of Chinese participants using open-ended questions to learn about the different aspects of self-love. Then, we conducted in-depth interviews with Chinese participants to explore the components of self-love. Because humans remain biased throughout the research process, even the most experienced researchers struggle to eliminate subjective experiences (Bashir et al., 2008). Therefore, trustworthiness (reliability and validity) must be considered (Golafshani, 2003). The coder agreement coefficient is usually used to evaluate the coding reliability in qualitative research. We used the coder agreement coefficient to evaluate the reliability of studies 1 and 2. The higher the consistency, the higher the reliability (Yang et al., 2006, p. 663; Zheng et al., 2010, p. 135). “Validity” represents whether people's real-life experiences can accurately be reflected in qualitative research. In Study 2, we adopted participant and nonparticipant test methods (McMillan and Schumacher, 2006; Fu et al., 2020).

## Study 1

### Materials and Methods

#### Participants

A purposeful sample was recruited on the Internet using the snowball sampling method. First, we published a poster of the recruitment questionnaire on the Internet and then asked participants, “Who else do you think is more suitable to answer?” or “Who do you think we should ask for more information?” After obtaining the participants' consent, the questionnaire poster was sent to them. Finally, we collected 109 questionnaires. Two of the questionnaires were carelessly answered, and one was answered by others and submitted twice, so these four questionnaires were regarded as invalid, leaving 105 valid questionnaires.

The participants came from nine provinces and three municipalities in the People's Republic of China. The sample was roughly gender-balanced, consisting of 47% men and 53% women; their ages ranged from 18 to 61 years (*M* = 32, *SD* = 11.5). The participants were very diverse, consisting of students, teachers, salespersons, nurses, the unemployed, etc. In terms of educational background, high school education accounted for 5.71%, university education accounted for 74.29%, and master's degree and above accounted for 20.37%. In addition, 48% of participants were unmarried, 4.7% were married with no children, over 47% were married with children, and 2.8% were divorced; no participant was widowed.

#### Procedure

The study was conducted between September 11 and October 19, 2018, after receiving the approval of Southwest University's ethics committee. During the period of questionnaire distribution, the researchers published a poster for the recruitment questionnaire on the Internet every day. The participants could scan the QR code or click the questionnaire link to answer through the questionnaire star (China's questionnaire collection website). The questions were as follows:

- “Do you think you are a self-loving person? What are the main manifestations?”
- “Do you know people around you who love themselves? What are their main manifestations?”
- “What do you think ‘self-love’ means in today's society?”

The participants had to answer the questions after reading and signing the informed consent form. The participants were asked to provide as much detail as possible and to write no <10 words in response to each question. Each participant was given 2 yuan as compensation.

#### Data Coding

First, we sorted and coded some of the answers. We used content analysis to analyze the text data of the answers through the following process: (1) reading and understanding the statements on self-love repeatedly; (2) establishing categories—based on the analysis of self-love-related statements, the principal investigator, through a discussion among psychology majors and experts and according to the different perspectives involved in the interpretation of self-love in the corpus, divided it into four dimensions: self, family, other, and society. A content analysis manual was developed, including the definition of each dimension, classification criteria, and typical examples. Then, we continued to collect answers. When new codes could not be generated anymore, saturation was considered to have been reached, and no more questionnaires were collected. Therefore, we obtained 105 responses about self-love. After that, we formed and trained the analysis group: a psychology doctoral student and two Chinese language master's students formed the analysis group; they used manuals and practice materials and discussed them with the group. The researchers of the analysis group independently classified and coded according to the analysis manual. In principle, each unit could only be classified into one category, and the frequency of classification for each category was recorded. Finally, we checked to see if there were any errors or duplications in the classification (Xue et al., 2020).

#### Trustworthiness

The coder agreement coefficient is usually used to evaluate the coding reliability in qualitative research (Zheng et al., 2010). The coder agreement coefficient was done through the following procedures. First, we calculated the mutual agreement of each of the two coders using the formula [L = 2M/(N_1_ + N_2_)] (where *M* stands for the number of completely agreed, *N*_1_ stands for the number of categories for the first coder, and *N*_2_ stands for the number of categories for the second coder), we calculated the three coders' mutual agreement degree separately and then averaged it. At this time, we got an “*L*.” Then, we applied L into the formula [*n* × (n/L)/[1 + (n−1) × (n/L)] (where *n* is the number of coders) (Yang et al., 2006, p. 664; Zheng et al., 2010), and we got the coder agreement coefficient.

### Results

#### Level 1: The Dimension of Self-Love

##### Self

In “self,” there were four main types of patterns. Among them, “cherish self” comprised 40% of the total items; it referred to cherish and respect oneself, protect oneself, take care of oneself, and improve oneself. For example, the participants answered, “Self-love is love of self, respect for self; take care of your body and emotions; make everything about you look your best and live happily ever after.” “Accept self” (6%) concerned “being able to accept the past self, being satisfied with the present self, and not being hard on oneself for the frustration of the outside world.” “Restrain self” (18%) referred to “managing your body, emotions, and personal life; not doing bad things, not doing things that damage your reputation or personality; having strict demands on yourself. Be a clean person.” “Persistent self” (11%) referred to “be yourself; in any condition to adhere to their own outlook on life, values, and worldview; have their own moral bottom line and principles.”

##### Family

Participants who described self-love also mentioned their family members (17%). How one treats family members is also a part of self-love; some answers included, “Don't be a burden on the family; care for the family and be responsible for them.”

##### Others

In the dimension of others (6%), respondents thought that they should treat and cherish others well. On the one hand, they should not embarrass or trouble others; on the other hand, they should care for, respect, and love others. Furthermore, because of their self-love, people around them would become better and more comfortable.

##### Society

In the Chinese public's view, society (18%) was an important part of self-love. Self-love meant realizing one's own value, not causing trouble to society, integrating into society, and respecting and contributing to society.

#### Level 2: The Reliability of Dimensions

The results showed that the coder agreement coefficient of each dimension (self, family, others, and society) was between 0.80 and 0.91.

### Discussion

The purpose of the content analysis was to explore the public's view of self-love in the context of Chinese culture. Three coders completed all the text data analysis independently. The coder agreement represents the coders coded the item into the same dimensions. The coder agreement coefficient was more than 0.80, which indicated that the content analysis had good reliability.

Self-love plays an important role in Chinese life. It involves the individual, relationships, and society. The findings are in line with previous research into the psychology of self-love in the Siku Quanshu and, at the same time, conform to the “*Ren*” in Confucianism. Generally, Chinese self-love includes not only self but also family and society. Confucius put forward “for Ren from self” (Chen and Xu, 2015) and “Be able to be yourself first, and then help those in need. This is the way to *Ren*” (Chen and Xu, 2015). The Chinese public's view includes not to drag down the family and to be responsible for one's family. Filial piety is an important concept in Confucian culture: “It's rare for a person to be filial to his parents and respect his elder brother but to enjoy offend the superior. There is no such person who doesn't like to offend but likes to make trouble. When a gentleman is committed to the foundation, and the foundation is established, the Tao comes into being. Filial piety is the root of *Ren*!” (Lun Yu·Xueer, p. 8). Confucian culture recognizes human self-love as the starting point and premise to love others.

This preliminary study revealed the general view of self-love in China: it accorded with the concept of “*Ren*” in Confucian culture, but a deeper understanding of self-love needed to be explored. Therefore, in the second study, we used in-depth interviews to investigate what self-love means to Chinese adults.

## Study 2

In Study 2, we aimed to explore what constitutes self-love. Therefore, we conducted a series of in-depth interviews with people of different ages and educational backgrounds to explore their inner world of self-love.

### Materials and Methods

#### Design

We adopted the Grounded Theory (GT) as our research method. GT is used to develop tentative theories and provides a set of clear and specific research steps. This method begins with the induction of data, through the process of iteration and comparison, until reaching data saturation (Stauss and Corbin, 1990). Data were collected through in-depth interviews. This approach provides a more relaxed atmosphere to collect detailed information and allows us to observe the participants' nonverbal expressions during the interview to understand Chinese interpretations of self-love (Boyce and Neale, 2006). Therefore, in strict accordance with GT procedure, we explored the connotations and structure of self-love using the information collected during the interviews.

#### Participants

We combined intensity sampling (based on the characteristics and functions of the sample itself to complete the research task) with snowball sampling (the researcher's own way of action); this is theoretical sampling in GT (Chen, 2000; Corbin, 2017). In the preinterview, seven Chinese college students from Southwest University participated. In the formal interview, we first published information about the candidates needed on the Internet and selected candidates who actively and enthusiastically signed up for the interview. In addition, through snowball sampling, these participants recommended people they thought to have the character of self-love and interviewees who could provide the largest amount of information. In order for this research to be considered valid, we needed to recruit at least 12 participants (Lincoln and Guba, 1985). After each interview, we started with open coding. When we found that there was no new opening code, the interview stopped. We eventually terminated recruitment after 18 interviewees (six males, 12 females). Their age ranged from 19 to 59 years (*M* = 31.59, *SD* = 10.25). Their educational background ranged from junior high school to doctoral degrees, and their professional identity included civil servants, university teachers, public institution staff, freelancers, college students, etc. They were mostly from the northeast of China.

#### Materials and Procedure

Preinterviews and formal interviews were conducted after receiving the approval of Southwest University's ethics committee. The participants read the informed consent form (Appendix 1), agreed, and signed it before the interview took place. They were informed that participation was voluntary and that they could withdraw from the study at any time. Interviews were audio recorded using the voice memo application on the researchers' mobile phones. The researchers obtained interview experience during the preinterview. Based on the results of the preinterview, we consulted experts and held a team discussion to determine the final formal interview outline.

The formal interview was arranged in the participant's free time. Each interview lasted from 45 to 70 min and took place in a quiet, comfortable environment (the school's counseling room, office, etc.). The formal interview outline included five questions: (1) Are you a person who loves yourself? (2) What are the main manifestations of self-love in life? (3) Are you surrounded by people who love themselves? (4) What are the aspects of your self-love? (5) What do you think of self-love in today's society? Each question contained the instruction “Please answer with specific examples.” Once the information obtained from the participants reached saturation, the interview ended. Each participant was given an incentive of 40 yuan or a gift of equal value for their participation. The interviews were conducted from March to June 2019.

#### Data Analysis

**Figure 1:**
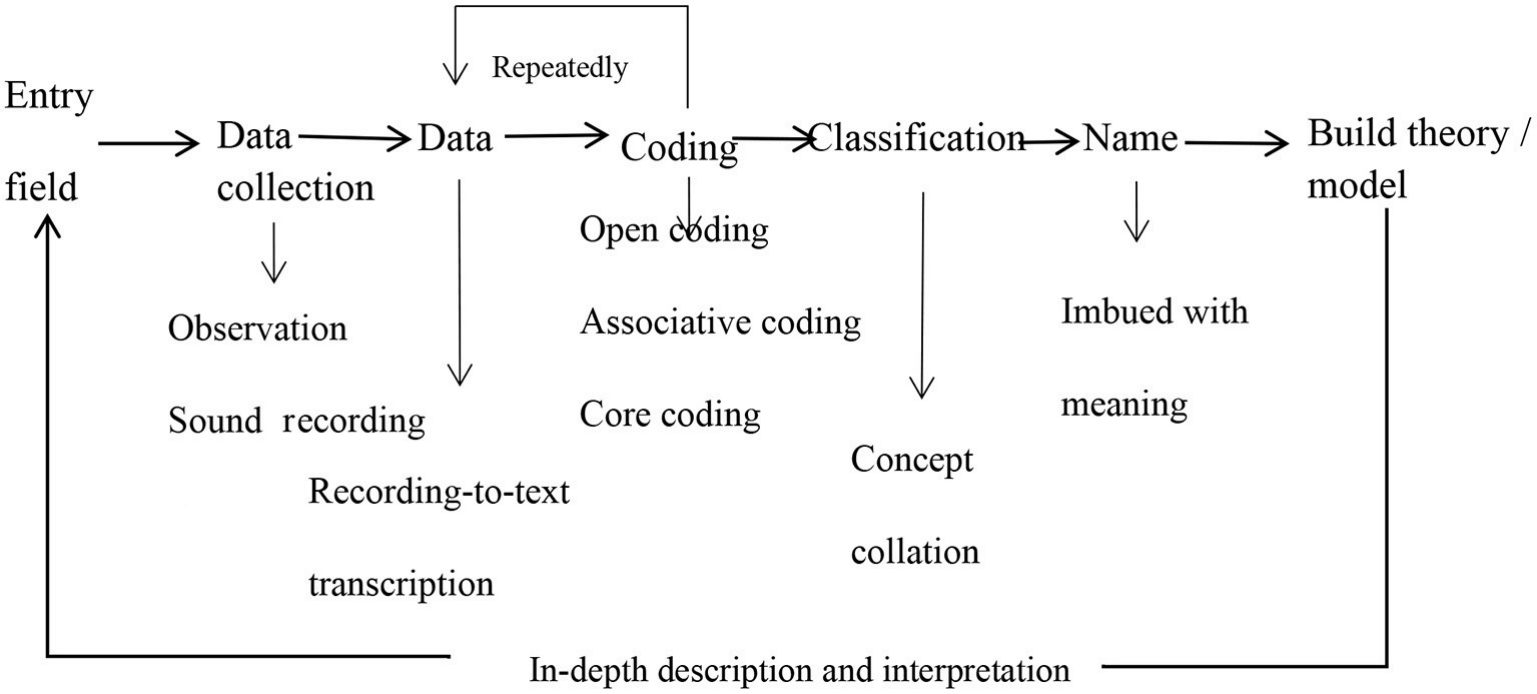
General flowchart of grounded theory (based on Wu and Huang, 2012).

The researchers transcribed the interview recordings into a Microsoft Word document as soon as possible after the interviews and carefully checked for errors and unclear phrases in the transcripts. After sorting the responses, each text file was between 7,000 and 17,000 words, with about 200,000 words overall. The text data were imported into the qualitative data processing software NVivo 11.0 and combined according to the general process of GT (Figure 1). Each participant was given a number according to the order of their interview (the number of participants ranged from z001 to z018). In the comparative text data, half sentences or whole sentences were classified into different nodes, and the text was encoded at three different levels according to the degree of abstraction (Stauss and Corbin, 1990).

In the open coding stage, sentences related to the meaning and expression of self-love were coded. Using the participants' unique expressions, we generated 315 distinct open codes, such as paying attention to health, not getting physically sick, cherishing one's roles, not relaxing, etc. In this stage of coding, the researchers adopted a completely open attitude and entered the original data freely and wholeheartedly without any theoretical framework (Chen, 2000; Corbin, 2017). According to our repeated reading of the interview text and the group discussion, we merged, refined, and sorted the data and clarified the internal relationships of each node (Fu et al., 2020) and combined them with the context at that time. The initial 315 open codes were summarized into eight categories: self-cherishing, self-acceptance, self-restraint, self-responsibility, self-persistence, foundation, necessity, and relevance. Through core coding, the relationships between each node were connected in series, and the preliminary connotation and structure of self-love were developed.

#### Trustworthiness

Reliability was improved by coder agreement coefficient and inquiry audit (Yang et al., 2006, p. 663). The coder agreement coefficient was the same as in Study 1, but the coding group was organized by two psychology postgraduates with qualitative research experience. “Inquiry audit” comes from the experience gained during the preinterview.

Validity was used in both participant and nonparticipant tests. In the participant test, the researcher selected three participants (all of whom had high school education or above) to provide feedback on the coding and conclusion; in the nonparticipant test, three coders with psychological training were invited to give feedback on the coding and conclusion.

### Results

#### Level 1: Components of Self-Love

According to the associative coding presented in Table 1, self-love consisted of five components: self-cherishing, self-acceptance, self-persistence, self-restraint, and self-responsibility.

**Table 1:** The connotation of self-love.

**Components (Associative code)**	**Subcomponent**	**Material sources/reference**	**Quote (open code)**
	Cherish self	13/63	*I have not considered why I should love myself, if I am good to myself, I am sure to feel more comfortable, and I can get happiness and satisfaction* (z007).
Self-cherishing	Protect self	10/23	*I'll ask her mother to tell her that girls should protect themselves from the outside world* (z010).
	Take care of self	17/130	*It might contain physiological, psychological and social, one's all-round care or one's maintenance* (z001).
	Respect self	7/12	*I think self-love should have a respect that is based on the premise of respecting others and respecting themselves, he will love himself more* (z014).
Self-acceptance	Accept one's real self	11/42	*I think self-love is very important, which means that I can fully accept myself as I am. Because this is very important because you are born like this, you can't choose how you are born. Can't choose* (z018).
	Develop oneself	8/22	*I think you usually cherish yourself, and you will make some choices, just like I just said that I will choose to practice calligraphy, and I want to learn something* (z003).
	Be ideal self	5/27	*I know that these choices I make can help me reach a higher level in the future, and I know what I meet in this area, these choices will also make me feel better in the future* (z003).
Self-persistence	Have a bottom line	6/12	*I feel that there is a bottom line; that is, when they pursue their dreams, they can't exceed their bottom line because of some interests* (z017).
	Have principles	6/16	*I'm a very principled girl, I think some things you do can't violate those principles, it's self-love* (z002).
	Have boundaries	4/4	*It turns out that these are manifestations of self-love, including sometimes talking about boundaries, grasping their own boundaries and not allowing others to cross them* (z005).
	Have judgment	8/15	*When I have conflicts with other people's opinions and ideas, I may have gone along with others' opinions before, but not now* (z006).
	Have dignity	5/15	*Because Chinese people have a traditional culture, I am not afraid of death. I'd rather die than lose my integrity. So, can you say they don't love themselves? On the contrary, they are very self-loving in our eyes* (z010).
Self-responsibility	Sense of responsibility	9/27	*Since you are a student, you should be able to deal with the word of students when you come, you should study and live, what you should do* (z011).
	Behavior of responsibility	10/54	*She is very responsible for her family: each member, her daughter, other people, and her relatives who have some relationship with him. And she provides guidance in their lives and careers* (z006).
Self-restraint	Conform to social norms	3/17	*I think a reasonable self-love respects human nature, but meets certain rules, but this rule will be beneficial to most people, or meet the requirements of social development* (z001).
	Prudence	10/41	*I think I didn't do anything wrong; I think I never cheated on the exam, and then I think she shouldn't cheat* (z002).
	Self-discipline	11/48	*I think there will be a quality of shendu. You may maintain your own open and aboveboard behavior, which may be more based on your self-love, the maintenance of order is also based on this self-love* (z007).

#### Self-Cherishing

This component was associated with 228 key words. It contains four subcomponents: cherish oneself, protect oneself, take care of oneself, and respect oneself. It shows people's positive self-feelings and actions toward themselves. Cherishing one's body, health, job, family, and so on were coded as “cherishing oneself”; protecting one's own safety, reputation, and dignity were described as “protecting oneself”; “take care of oneself” appeared most frequently, including being kind, adapting, and paying attention to and satisfying oneself; the last components included respecting oneself and others and respect from others.

#### Self-Acceptance

This component was mentioned 91 times, and it means people accept their real selves, and they are committed to making themselves better and ultimately achieving their ideal self. A clear understanding and acceptance of oneself was ascribed to “accepting one's real self,” which appeared most frequently. Another category, “improve oneself” included constantly improving, acquiring new abilities, and learning until finally becoming what one wants to be.

#### Self-Persistence

Self-persistence suggested that people adhere to their own beliefs and do not compromise when dealing with people; this component was mentioned 62 times. “Have principle” indicated that individuals have their own principles when they do things and cannot violate them. “Love your country and nation” indicated that individuals have inviolable identities and the integrity of being humans, which was associated with “have dignity.” “Have judgment” referred to the individuals' independence, respect, and adherence to their own ideas. “Have a bottom line” meant that when people do things, they cannot go below their minimum threshold.

#### Self-Responsibility

Self-responsibility suggested that self-love is the individual's own responsibility and obligation, which consisted of two subcomponents: sense and behavior. It appeared 76 times. “Sense of responsibility” referred to playing one's own role in life, the responsibilities of different identities, and knowing what one should and should not do. In life, the responsibility to implement practical actions is more frequent, including trying one's best, doing a good job, taking good care of one's family, and living by one's own values.

#### Self-Restraint

Self-restraint suggested that individuals consciously constrain their behaviors according to societal expectations (such as law and morality). This category consisted of social norms, prudence, and self-discipline, which came up 105 times. Self-love meant that an individual could conform to basic social requirements (do not cheat, do not do bad things, do not take drugs, etc.), which could be summed up as “prudent behavior.” Self-discipline meant to consciously live normally and restrain oneself without the supervision of others.

In general, self-love was not only about loving self, but it was also closely related to loving others. From the original data, it was found that 12 participants mentioned loving oneself 25 times. In addition, when talking about self-love, most of the participants mentioned the relationship between self-love and loving others, such as “Self-love, in fact, I think another level is to love others, which should also be undertaken; I think if we say that the realm is a little higher, it may also include that it is better to love others” (z014).

**Figure 2:**
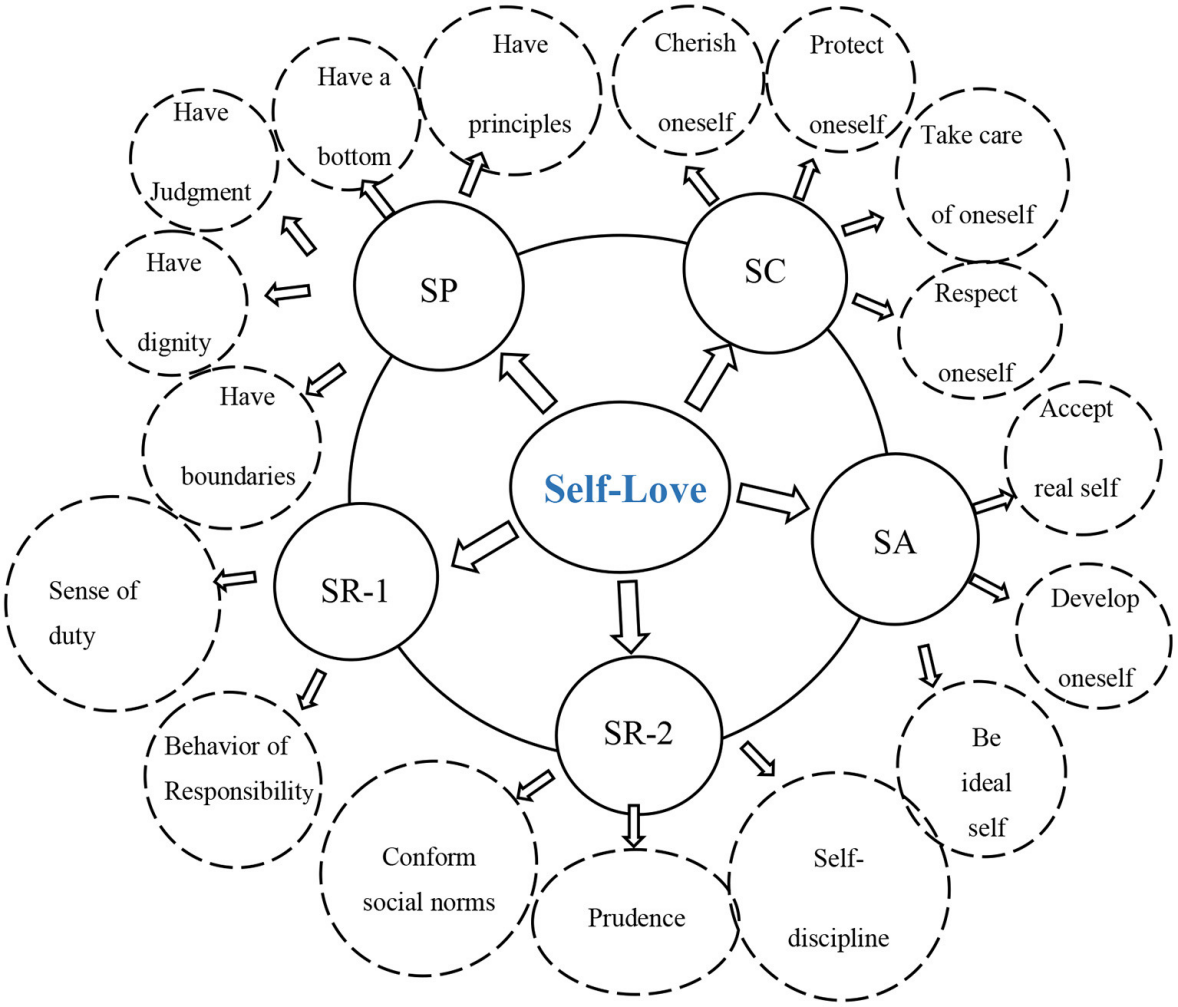
The connotation of self-love. SA, self-acceptance; SC, self-cherishing; SP, self-persistence; SR-1, self-responsibility; SR-2, self-restraint.

Based on the above analysis, it can be summarized that (1) self-love mainly included five components: self-cherishing, self-acceptance, self-persistence, self-responsibility, and self-restraint; and (2) self-love was not equivalent to loving oneself but was closely related to loving others (Figure 2).

### Level 2: The Relationship Characteristics and Structure of the Five Components

Based on the aforementioned findings regarding the five components of self-love, a comparative analysis was used to explore the relationship between these components. After repeatedly returning to the original materials for induction, this study found a correlation between the five components of self-love. In addition, self-love and self, interpersonal, and social harmony exhibited characteristics in common.

#### Foundation

In Table 1, self-responsibility had a sense related to taking responsibility for oneself. In Table 2, the results illustrated that self-responsibility was the basis of self-restraint. One participant thought that self-love was the embodiment of responsibility and lust. He narrated controlling lust by being responsible in life.

**Table 2:** The relationship and structure of self-love.

**Associative code**	**Quote**	**Material sources/reference**
Foundation	*Self-love, I think it is now a manifestation of responsibility and lust, which is reflected by these two aspects; If a person has lust in his heart, he can control his lust, and then his sense of responsibility is strong, so that he is a man in our traditional sense of self-love* (z010).	1/7
Necessity	*Some people just don't cherish their bodies, feel like staying up late, or do something that is not good for their health. I don't think this is self-love* (z002).	13/27
	*People all have lust, which is inevitable, which is normal. Even animals have lust, not to mention human beings. But you should control it* (z010).	1/5
	*I haven't analyzed what I am like, and I don't care much about other people's ideas, so I don't think about whether I should change. I always feel that I live according to my heart* (z013).	6/11
Integrity	*In fact, I would like to say that these three aspects may actually be the three dimensions of physiology, psychology, and society, a cherishing or a maintenance of all aspects of oneself* (z001).	7/18

#### Necessity

Self-restraint was a necessary but not sufficient condition. Six participants mentioned that self-love involves a kind of constraint, which reflects a necessary condition of self-love. As the participants mentioned, “In fact, the universal sense of self-love makes me feel a little constrained” (z016) and “It is a restriction of the self” (z014). However, the content of self-restraint embodied different standards from that of individual self-restraint. It can be said that self-restraint was not a sufficient condition for self-love.

According to the participants, self-cherishing restrained them from doing bad things. When the participants mentioned cherishing their bodies, they stated they would not harm their bodies. Protecting oneself meant not doing things that harmed one's dignity. “Take care of oneself” meant controlling stress, not feeling forced into decisions, and adapting to prevent emotions from getting out of control.

Self-love could help people accept their own lust and/or control it. One participant reported that he did not fool around with his classmates. He thought he resisted lust and therefore loved himself.

Finally, six participants mentioned that self-persistence would restrain them from doing things beyond their bottom line, boundaries, violating principles, or damaging the national system.

#### Integrity

**Figure 3:**
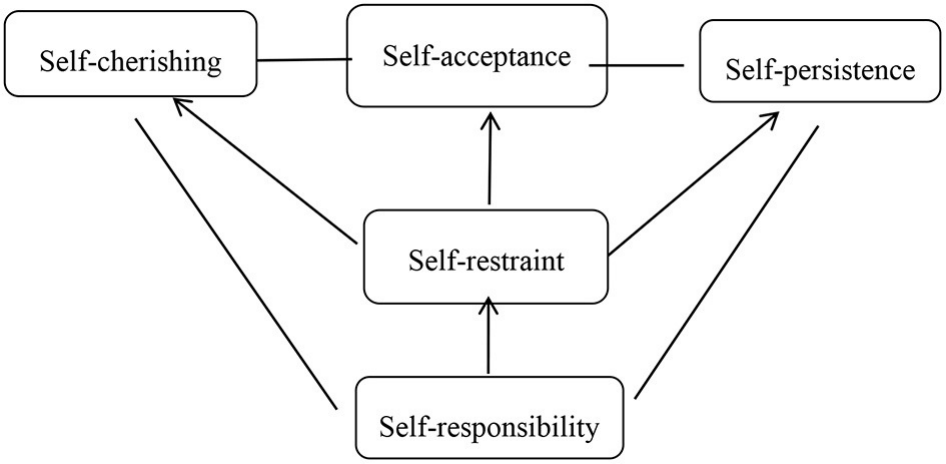
The structure of self-love.

The results of this study suggested that Chinese people's understanding of self-love is presented as a comprehensive whole in the form of self-cherish, self-acceptance, self-restraint, self-responsibility, and self-persistence in every aspect of their lives. The integrity of personality meant that, although personality has multiple factors and characteristics, they are not isolated in real people but closely related and integrated into an organic organization (Huang, 2002). In conclusion, these five factors were integrated in the person with self-love (Figure 3).

#### Mutuality

At the same time, the participants mentioned the relationships among self, family, others, and society. The relationships among self, interpersonal relations, and social harmony had the same direction. As mentioned by the participants, in terms of relationship with the self, “Internally, if I do self-love, I will feel that I also have this ability, which is a manifestation of self-development, or I can get some sense of achievement or value from it” (z001). In terms of relationship with the family, “In this kind of environment, I am stretching because the surrounding environment is like this. If my family is with me, it is also a feeling of stretching. This family must be very harmonious, and the atmosphere is very good” (z005). In terms of relationship with others and society, “In fact, in this society, if we all love ourselves, believe in ourselves, cherish our reputation, and don't care what others see, we may make a lot of contributions to the development of society” (z002).

### Level 3: Model and Testing of Research Results

#### Reliability Test

The reliability test still used consistency reliability. After reviewing the original data, we invited two graduate students majoring in psychology to code 50% of the interview's texts each (*n* = 9) and finally calculated the coefficient of conformity with the researcher's classification. The final reliability value was 0.89.

#### Validity Test

We assessed the examination method for participants and nonparticipants. After obtaining the preliminary results, the researchers shared the coding and conclusions with the participants and nonparticipants to test the descriptive and analytic validity of the data.

In the participant test, the researcher selected three participants (all of whom had a high school education or above) to provide feedback on the coding and conclusion, including one university teacher, one master's student, and one freelancer. The participants were required to review the interview content and combine the interview results to ensure the accuracy of the expressions in the interview research. In the nonparticipant test, three people with psychological training were invited to give feedback on the coding and conclusion. One was a psychology professor at Shenyang Normal University and two were doctoral students in psychology. The feedback from six inspectors was summarized, examined, and dealt with in detail (Appendix 3). In addition, we checked the nonverbal information of each participant and the recorded keywords to compare with their verbal information.

### Discussion

Study 2 explored the psychological components of Chinese self-love using GT, and we found that Chinese self-love includes self-cherish, self-acceptance, self-restraint, self-responsibility, and self-persistence; and these five components are interrelated and form a stable personality structure.

Compared with Study 1, there are several points to be noted. First, the factors used in Study 1 were repeated in the interview: cherishing self, accepting self, restraining self, and persisting self, which represented the public's view of self-love. At the same time, the results of Study 2 elaborated the content of Study 1.

First, the results of Study 2 did not reflect the four dimensions (self, family, others, society) shown in Study 1; in fact, the interviewees included family, others, and society into their self-concept. This may be because the participants' answers were more detailed and clearer during the interviews.

Second, these components are based on the Confucian culture. They indicate that self-love is how a man demands self-cultivation through his conscience or moral sense. “Self-responsibility” is also a fundamental component of self-restraint. It is in line with the Confucian culture stressed by the *Neishengwaiwang* (internal saints and external kings), “To the world as their own responsibility” of “*Junzi”* personality. “Self-restraint” is the core component of self-love. As well as the Confucian culture advocates taking the individual as the starting point, through the subjective cultivation of “honesty” and “respect,” “Unity of knowledge and practice,” they internalize this as a moral principle to restrain themselves and supplemented by external punishment, to achieve the responsible personality of “restrain self and give others more convenience” (Ling et al., 2003; Ren, 2008). This also reflected in other components; this is the reason “take care of your body and emotions” is considered part of the “self-cherish” category, but “managing body, emotions, and personal life” is considered part of the “self-restraint” category. Others such as “self-acceptance,” people accept their own desires, but also to restrain their own desires. Confucian culture is to realize the “*Ren”* of oneself with the practical spirit of “deny self and return to propriety” (Chen and Xu, 2015). In addition, modern Chinese are influenced by Confucian culture, but they are also developing. People began to put more emphasis on independence and autonomy, adhere to their own beliefs, and do not compromise. This may be an increasingly popular trend of individualism in modern Chinese society (Huang et al., 2018).

Third, self-love is an interrelated and integral personality structure. Since ancient times, self-love has been an important part of the ideal personality of Confucian culture, and it is also the basis of sound personality in modern times. When participants described self-love, they first mentioned holistic self-love and then described its behavioral characteristics. They also emphasized the relationship between the behaviors typical of self-love.

## General Discussion

This study explored Chinese self-love from two perspectives. Dimensions and components. The results of the internal consistency reliability tests showed that the two qualitative studies were strong. Based on the background of Chinese culture and data collected through open-ended questionnaires, this study showed that Chinese people's understanding of self-love is expressed in four dimensions. Through the in-depth interviews, we delved deeper into the connotations and psychological structure of Chinese self-love by using GT as our analytical method. Based on these results, we conclude that Chinese self-love is a complex personality structure dominated by Confucian culture and that it is a guide for Chinese people to choose what kind of person one should be (Wang, 2012).

From the results, we conclude that Chinese self-love is still society-oriented. It is in line with self-love being society-oriented that the harmony, tranquility, and order of the society in Confucian culture are achieved by individual efforts. As the “*Daxue*” wrote, “Cultivate one's moral character, complete one's family, govern one's country and bring peace to the world.” In the open-ended questionnaire survey, we found that the respondents thought that self-love included the dimensions of others and society. People are more likely to include their family members in their self-concept and to associate themselves, family, and society with others. The idea that Chinese self-concept includes family and friends has long been known (He and Zhu, 2010). Further, the results of the second study show that self-harmony, family harmony, and social harmony are closely related. In addition, self-responsibility is an important component of self-love. These results also reflect the differences in the understanding of self-love between Chinese and Western cultures. Chinese people's understanding of self-love is based on self-responsibility and centered on self-restraint; the Chinese concept views individuals as embedded in a network of social relationships. Personal self-love cannot cause trouble to others but can also make the whole society better. However, self-love in Western culture tends to not reflect social orientation but to focus more on the self. Such as the research involving female college students who had experienced sexual violence, self-love was described as feeling “comfortable” and setting aside time to nurture self, etc. (Sinko et al., 2019). Tokwe and Naidoo (2020) concluded self-love is one of the journeys toward a new normal experienced; it could make the person who lived experiences of human immunodeficiency virus take ownership of diseases. Therefore, it is clear that people's understanding of self-love is different between Chinese culture and other cultures.

## Limitations of the Present Study

Our research has limitations. First, the collected data consisted of participants' self-reports. In general, self-report methods are easily influenced by the participant's desire for social approval. More importantly, we used a small sample to gather exploratory data; therefore, the results are not generalizable to the Chinese population at large. In addition, gender, age, and occupation were not strictly controlled in the study. Women seem to be more interested in this topic, and individuals of different ages and occupations might have different understandings of self-love; thus, controlling for these variables might yield different results. Considering the diversity of self-love in daily life, this was an initial study that revealed an effective way to collect further data on self-love. Future studies conducting experimental tasks might provide relevant information about the construct of self-love.

## Conclusion

As stated in the *Introduction*, this study sought to understand Chinese people's understanding of self-love in both breadth and depth, to provide a reference point for psychological research into self-love, and to provide evidence for cross-cultural differences in the understanding of self-love. To accomplish these aims, this study sought to explore Chinese people's understanding of self-love in both breadth and depth through two qualitative researches. We found that the Chinese view of self-love includes four dimensions: self, family, others, and society. In the real world, we found that the connotations of self-love comprise five components: self-cherishing, self-acceptance, self-restraint, self-responsibility, and self-persistence; these components help form the structure of self-love. This study provides a reference point for self-love in Chinese culture, which may be used for future psychological and cross-cultural studies.

## Geolocation Information

This study was conducted at Southwest University (106.43 degrees east and 29.82 degrees north).

## Data Availability Statement

The raw data supporting the conclusions of this article will be made available by the authors, without undue reservation.

## Ethics Statement

The studies involving human participants were reviewed and approved by Southwest University's ethics committee. Written informed consent to participate in this study was provided by the participants' legal guardian/next of kin. Written informed consent was obtained from the individual(s) for the publication of any potentially identifiable images or data included in this article.

## Author Contributions

LX: Writing – original draft preparation, investigation, and data analysis. XH: project administration, supervision, and funding acquisition. NW: writing – reviewing. TY: funding acquistion. All authors: contributed to the article and approved the submitted version.

## Conflict of Interest

The authors declare that the research was conducted in the absence of any commercial or financial relationships that could be construed as a potential conflict of interest.
