# Supplementary material for: A Qualitative Exploration of Chinese Self-Love
Source: Front Psychol. 2021 Mar 29;12:585719. doi: 10.3389/fpsyg.2021.585719 (PMC8040951; doi:10.3389/fpsyg.2021.585719)
Supplement: Supplementary file 1 [file Data_Sheet_1.pdf]

Appendix 1 ： Informed consent

The researcher is a Ph.D. student majoring in psychology at Southwest University. At present, I am carrying out research on the topic of "the character of self-love of Chinese adults".The purpose of this study is to understand what self-love means to adults in the context of Chinese culture? How to develop the character of self-love? What is the function of self-love? I hope that through your personal experience and sharing can assist researchers in their research. If you agree to participate in this in-depth interview, you will have the following rights and obligations:

1. I agree to accept this in-depth interview. The time of each interview is about 50-60 minutes, and I will cooperate with the researcher to increase or decrease the time according to the actual situation of the interview.

2. In order to avoid the omission of materials or misunderstanding of researchers, I agree that the researchers record the interview contents in the way of recording and notes during the interview process, and agree to transcribe the recording into words for analysis and research; and I am willing to assist the researchers in the confirmation and correction of the semantics of the manuscript, and cooperate with the follow-up necessary.

3. This interview is anonymous. I understand that all personal data in this study are handled in accordance with the principle of confidentiality, and the researcher will never disclose it to the outside world. After the completion of the study, all data will be kept by the researcher or handed over to me.

4. I have understood the purpose of this study and I am willing to actively share personal experience. At the same time, in the interview process, I have the right to decide the depth and breadth of the content to be shared, and I have the right to retain the part that I don't want to disclose.

5. At the end of the study, I have the right to know the complete results of the study, and I can ask the researcher and get explanations at any time if I have any questions about the researcher.

I have understood my rights and obligations, and I agree to participate in this study.

Participant signature: Researcher signature:

Contact information of participants: Contact information of Researcher:

Appendix 2: Interview record sheet

Form of interview: Interview time:

Age:

Gender:

Nationality:

Occupation:

Monthly income:

Education background:

□ High school or below □ University □ Master degree or above

Marital status:

□Unmarried □Married and unfertilized □Married with children □Divorce

□Widowed spouse

Family situation (e.g. brothers and sisters or raising children):

The special aspects in the expression and language of the participant:

Problems to be noted and supplemented:

Participant's own feelings:

Appendix 3: Validity Feedback and Treatment

Source of feedback	Original research results	Feedback	Handling of feedback
Participant	Cherish your body	*Health, including physical and mental health. The relationship between cherishing one's own body and cherishing one's health is suggested to be reconsidered* (YY).	Cherishing one's own body also includes the problems of not harming one's own body and chastity, while cherishing one's health includes physical health and mental health, which are still handled separately.
	I don't think I'm going to work hard now. Don't affect others. You just must go to your last post safely and retire for more than a year.	*Prefer to express the feeling of sloppy work, not to do the current job with all one's strength*(SJ); *I don't think that's an expression of responsible behavior. It may also be a low responsibility behavior*(YY)	After returning to the original materials and reading them repeatedly, remove this item.
Non-participant	Love your nation and country	*I think it should be put into a sub-component of dignity* (W, G)	Go back to the original material and read it again and discuss it with relevant researchers to classify this

		article as self-persistence （have dignity）
conscientiousness	Responsibility cognition and conscientiousness are mixed and inseparable (G, L)	After reconsidering and discussing, the responsibility cognition and conscientiousness are reclassified. It is divided into the cognition of responsibility and the behavior of taking responsibility, and renamed.
